# Characteristics of coronary artery disease in chronic kidney disease

**DOI:** 10.1007/s10157-019-01718-5

**Published:** 2019-03-04

**Authors:** Hideki Fujii, Keiji Kono, Shinichi Nishi

**Affiliations:** Division of Nephrology and Kidney Center, Kobe University Graduate School of Medicine, 7-5-2, Kusunoki-cho, Chuo-ku, Kobe, Hyogo 650-0017 Japan

**Keywords:** Cardiovascular disease, Coronary artery disease, Chronic kidney disease

## Abstract

Patients with chronic kidney disease (CKD) commonly experience cardiovascular disease (CVD), and a major cause of death in these patients is CVD. Therefore, the prevention of CVD progression is very crucial in patients with CKD. Recently, this relationship between CKD and CVD has increasingly been examined, and a concept termed “cardiorenal syndrome” has been advocated. Coronary artery disease (CAD) and myocardial injury are crucial factors that contribute to the occurrence of CVD. The initial step CAD is endothelial dysfunction that can be detected as a decrease in the coronary flow reserve (CFR). The previous studies have reported that decreased CFR is significantly correlated to coronary events and mortality. Furthermore, CFR reduces with a decline in the kidney function. Another important presentation of CAD is coronary artery calcification. Vascular calcification is a crucial pathophysiological state, particularly in patients with CKD, and it affects the stability of coronary atherosclerotic plaque. In CKD, not only the traditional risk factors but also CKD-related non-traditional risk factors play key roles in CVD progression. Therefore, the mechanisms responsible for CVD progression are very complex; however, their clarification is crucial to improve the prognosis in patients with CKD.

## Introduction

Chronic kidney disease (CKD) has recently been gaining attention, because many studies have demonstrated a strong correlation of CKD with mortality. Cardiovascular disease (CVD) is a well-known major cause of death in patients with CKD. Therefore, understanding the association between CKD and CVD is crucial. To this end, the concepts of “cardiorenal association” and “cardiorenal syndrome” have been advocated [1], and a large number of clinical and experimental studies have attempted to elucidate the detailed mechanisms. Furthermore, CKD is considered to be a crucial risk factor for CVD even in the field of cardiology [2].

Along with the classical risk factors such as hypertension, smoking, hyperlipidemia, diabetes mellitus, and aging, CKD-specific non-classical risk factors contribute to CVD progression in CKD. CKD-specific non-classical risk factors include inflammation, anemia, volume overload, oxidative stress, renin–angiotensin system, sympathetic nerve system, uremic toxins, and chronic kidney disease-mineral bone disorder (CKD-MBD). As mentioned above, many studies have assessed the cardiorenal association; however, the detailed pathophysiological mechanisms remain unknown.

**Fig. 1:**
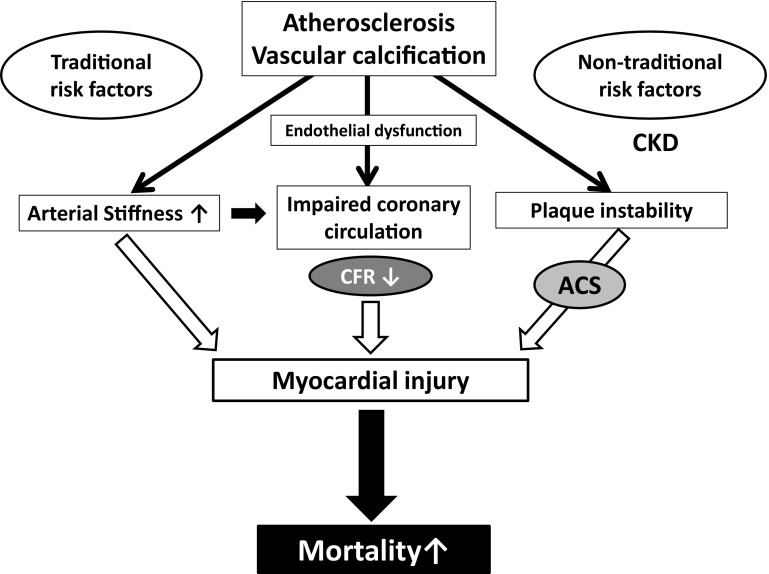
Putative mechanisms of CAD in CKD. *CAD* coronary artery disease, *CKD* chronic kidney disease, *CFR* coronary flow reserve, *ACS* acute coronary syndrome

One of the crucial pathophysiological manifestations of CVD in CKD is coronary artery disease (CAD). The CAD prevalence and the number of severe coronary artery stenoses increase as kidney function deteriorates [3, 4]. However, several aspects regarding this subject including the pathophysiological mechanisms remain unclear. In the present review, we summarize the characteristics and possible pathophysiological mechanisms of CAD in CKD based on the present clinical and experimental evidences (Fig. 1).

## Prevalence and clinical characteristics of CAD in CKD

A previous study conducted in the United States has reported that the averaged estimated glomerular filtration rate (eGFR) in 14,527 patients with acute myocardial infarction (AMI) was 70 ± 21 mL/min/1.73 m^2^, and 33.6% of these patients had CKD [5]. A Canadian study using a large population cohort demonstrated significantly higher incident AMI rates in patients with CKD than in those with diabetes [6]. Data of Japanese patients undergoing hemodialysis showed that the cause of death was AMI in 3.0% of these patients [7]. Moreover, based on the data of patients with non-dialyzed CKD and those undergoing hemodialysis in the United States, although the AMI prevalence increased with decrease in the eGFR, it decreased in patients undergoing hemodialysis [8, 9].

Several studies have evaluated the presence of CAD in asymptomatic patients new to hemodialysis [10, 11]. These data demonstrated that approximately 50% of these patients already had CAD without any clinical symptoms. However, these studies were performed during the first decades of the 2000s and a recent study has reported that the CAD prevalence has decreased among these patients in recent years [12].

The diagnosis of CAD in patients with CKD is very challenging, because these patients do not demonstrate the typical clinical symptoms of CAD and do not show the typical changes observed in CAD on electrocardiogram (ECG), such as ST-T change and abnormal Q wave. An interesting study has compared the clinical symptoms of patients undergoing hemodialysis with those not undergoing hemodialysis [13]. The percentage of those with chest discomfort, those with ST-T changes, and those with an AMI diagnosis at the emergency department visits was significantly lower, and the percentage of those with pulmonary edema, cardiac arrest, and death during hospitalization was significantly higher in the hemodialysis group than in the non-hemodialysis group. Furthermore, patients with AMI who had lower kidney function had poor prognosis [5]. Thus, the presence of CKD is a crucial issue for patients with CAD.

## Endothelial dysfunction of coronary artery

Endothelial dysfunction is the first step of atherosclerosis, and albuminuria is believed to reflect endothelial dysfunction. Endothelial dysfunction evaluated using acetylcholine-stimulated forearm blood flow was significantly associated with CVD in patients with hypertension [14]. Moreover, not only decreased kidney function but also albuminuria is reportedly associated with an increased CVD risk [15, 16].

**Fig. 2:**
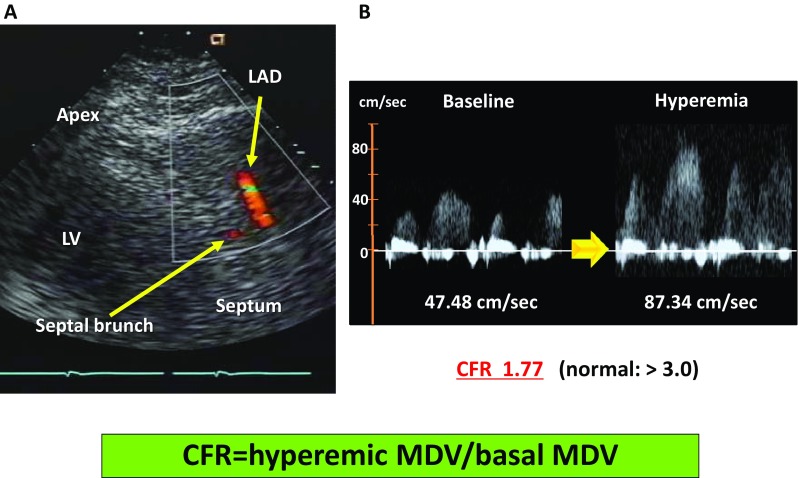
Measurement of CFR. **a** Visualization of the coronary artery using transthoracic echocardiography. **b** Coronary flow velocity at baseline and during hyperemia in patients with abnormal CFR. **a** Imaging of LAD using transthoracic echocardiography. First, the MDV at baseline is measured; thereafter, the MDV during a hyperemic condition induced by ATP administration (140 µg/kg/min IV) for 3–5 min **b** is measured. CFR is calculated as the ratio of hyperemic MDV to basal MDV. *CFR* coronary flow reserve, *LAD* left anterior descending artery, *MDV* mean diastolic velocity

**Fig. 3:**
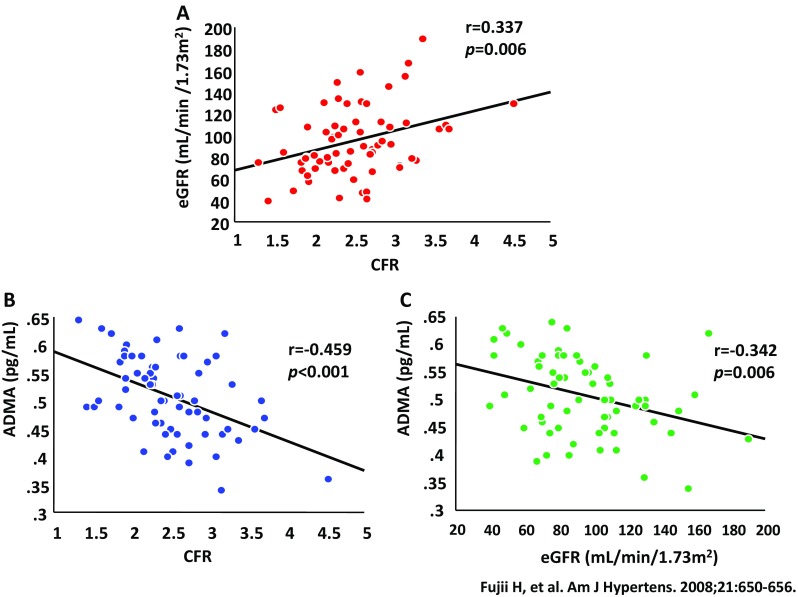
Relationship among eGFR, CFR, and ADMA. **a** Relationship between eGFR and CFR. **b** Relationship between ADMA and CFR. **c** Relationship between ADMA and eGFR. *eGFR* estimated glomerular filtration rate, *CFR* coronary flow reserve, *ADMA*, asymmetric dimethylarginine

Impaired blood flow in the small intramural resistance vessels or in the coronary capillary system that cannot be visualized using coronary angiography results in decreased coronary microcirculation [17]. Coronary blood flow normally increases automatically from the resting level to the peak level in response to increases in the myocardial oxygen demand [18]. Such a change in the coronary blood flow is regarded the coronary flow reserve (CFR). Several studies have demonstrated that CFR is significantly associated with kidney function [19–21]. Several our clinical studies assessed CFR using transthoracic Doppler echocardiography (Fig. 2) [20, 22–24], a non-invasive and safe method that does not involve the risk of radiation exposure. Our data showed that CFR was significantly associated with the estimated glomerular filtration rate (eGFR) in hypertensive patients without a significant coronary artery stenosis (Fig. 3a) and that both CFR and eGFR were significantly associated with asymmetric dimethylarginine (ADMA), an endogenous competitive inhibitor of nitric oxide (NO) synthase (Fig. 3b, c) [20]. Therefore, decreased local NO production following an increase in ADMA may lead to impaired microcirculation in the kidneys and heart, particularly in CKD. In fact, the previous studies have reported that decreased CFR was related to mortality in patients with CKD not undergoing hemodialysis as well as in those undergoing hemodialysis [25, 26].

## CAC in CKD

There are several risk factors for coronary artery calcification (CAC): age, diabetes mellitus, and CKD are believed to be the most important of these [27, 28]. With population aging, the CAC prevalence is increasing and male sex has a greater impact on CAC [29]. Furthermore, racial differences are also observed with respect to CAC progression, with Asians reported to have a lower prevalence than in the Western countries, including United States [30]. The CAC prevalence is much lower in Japan than in the other countries. Decreased kidney function is an important risk factor of CAC, and it has been reported that the prevalence of severe CAC increases with worsening of kidney function [31]. At the initiation of hemodialysis therapy, 81% of the study subjects in our earlier study already had CAC; 43.8% had mild-to-moderate CAC, and 37.5% had severe CAC [32]. The CAD prevalence is higher in patients with CAC and the prevalence is related to CAC severity [33]. This finding is also supported by the results of several studies conducted on only patients with CKD [31, 34].

Vascular calcification is commonly observed in CKD, because, in addition to several classical risk factors, patients with CKD also have certain unconventional risk factors of vascular calcification [28]. Among the various risk factors, mineral bone disorder is believed to be the most crucial factor for patients with CKD. The underlying mechanisms include the role of elevated serum phosphate levels, parathyroid hormone levels, and fibroblast growth factor 23 levels as well as decreased active vitamin D and klotho. Although these factors exert a considerable influence on the progression of vascular calcification in CKD, phosphate is the most important factor [28, 35]. The supposed mechanisms of vascular calcification involve the transformation of vascular smooth muscle cells into osteoblast-like cells by the uptake of phosphorus into cells through sodium-dependent phosphorus co-transporters and decrease of inhibitors against vascular calcification [35]. Even in the general population, serum phosphate levels are significantly associated with CAC prevalence [36]. Serum phosphate levels are also significantly associated with not only increased CAD, but also increased the other CVD events [37, 38]. Furthermore, the results of a meta-analysis have demonstrated that the presence of vascular calcification is significantly associated with higher CVD events and mortality [39].

## Impact of CAC on CAD in CKD

The pathophysiology of vascular lesions related to CAD is broadly classified into atherosclerosis and arteriolosclerosis.

American Heart Association has reported the Stary classification for atherosclerosis of coronary artery (Table 1) [40]. As per this classification, advanced types of atheroma, such as type 4 and 5, include calcific lesions. As mentioned above, CAC is a very important and prominent vascular lesion for CKD. There are two types of vascular calcifications based on the mechanisms of vascular calcific formation: one mechanism involves atherosclerotic calcification occurring in the intimal layer of the artery, while the other involves Monckeberg’s arteriosclerosis that occurs in the medial layer of the artery. Although these two types of vascular calcifications can be observed in patients with CKD, Monckeberg’s arteriosclerosis is often more prominent. With the progression of the stage of CKD, vascular calcification progresses and is a very crucial clinical problem, particularly in patients with CKD stage 5D (those undergoing dialysis).

**Table 1:** Stary classification Stary et al. [40]

Classification	Histology	Main mechanisms of progression
Type I (initial lesion)	Increase in macrophages and formation of scattered macrophage foam cells	Lipid accumulation
Type II (fatty streak lesion)	Mainly intracellular lipid accumulation	Lipid accumulation
Type III (intermediate lesion)	Type II + extracellular lipid pools	Lipid accumulation
Type IV (atheroma lesion)	Type II + extracellular lipid core	Lipid accumulation
Type V (fibroatheroma lesion)	Lesion with lipid core, fibrous cap, and calcification	Increase of VSMC and a fibrous component
Type VI (complicated lesion)	Lesion with ulceration, hemorrhage, and thrombus	Hemorrhage, thrombus, and hematoma

*VSMC* vascular smooth muscle cell

**Fig. 4:**
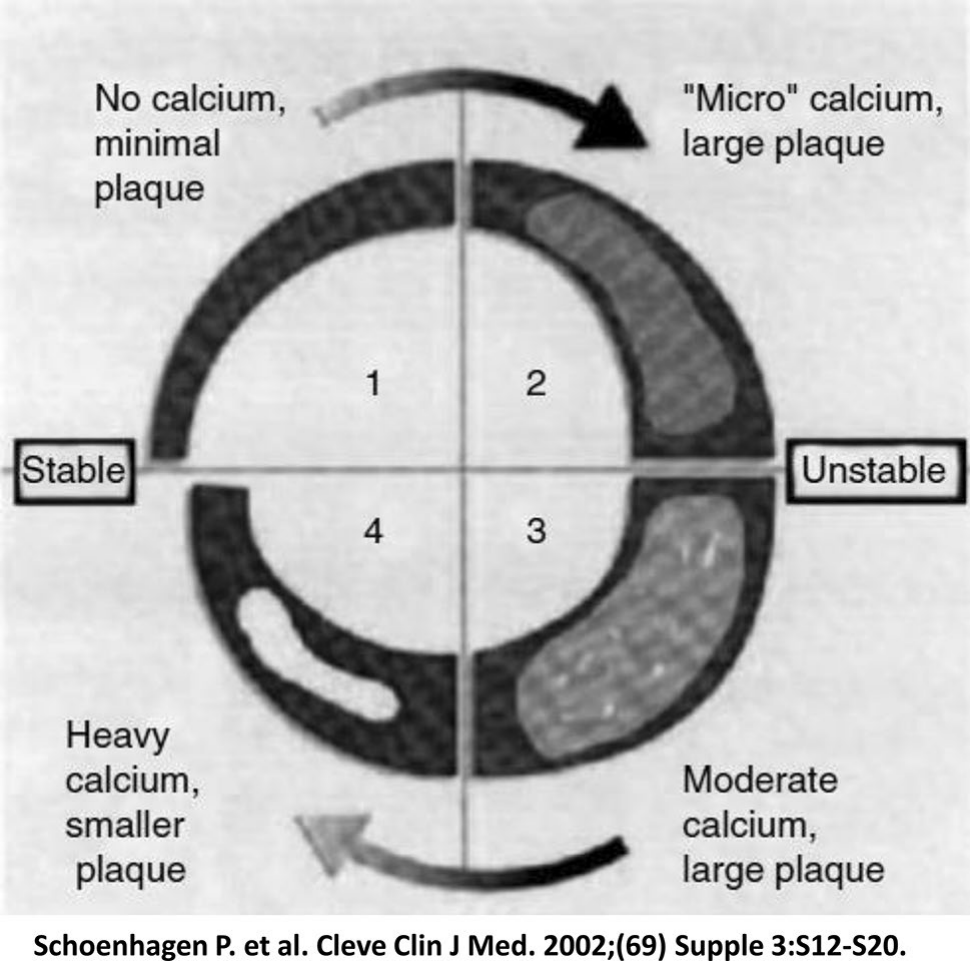
Impact of the degree of vascular calcification on plaque stability. Coronary artery calcification alters plaque stability depending on the size and distribution of the calcium deposit. Spotty calcification is strongly associated with the occurrence of acute coronary syndrome

**Fig. 5:**
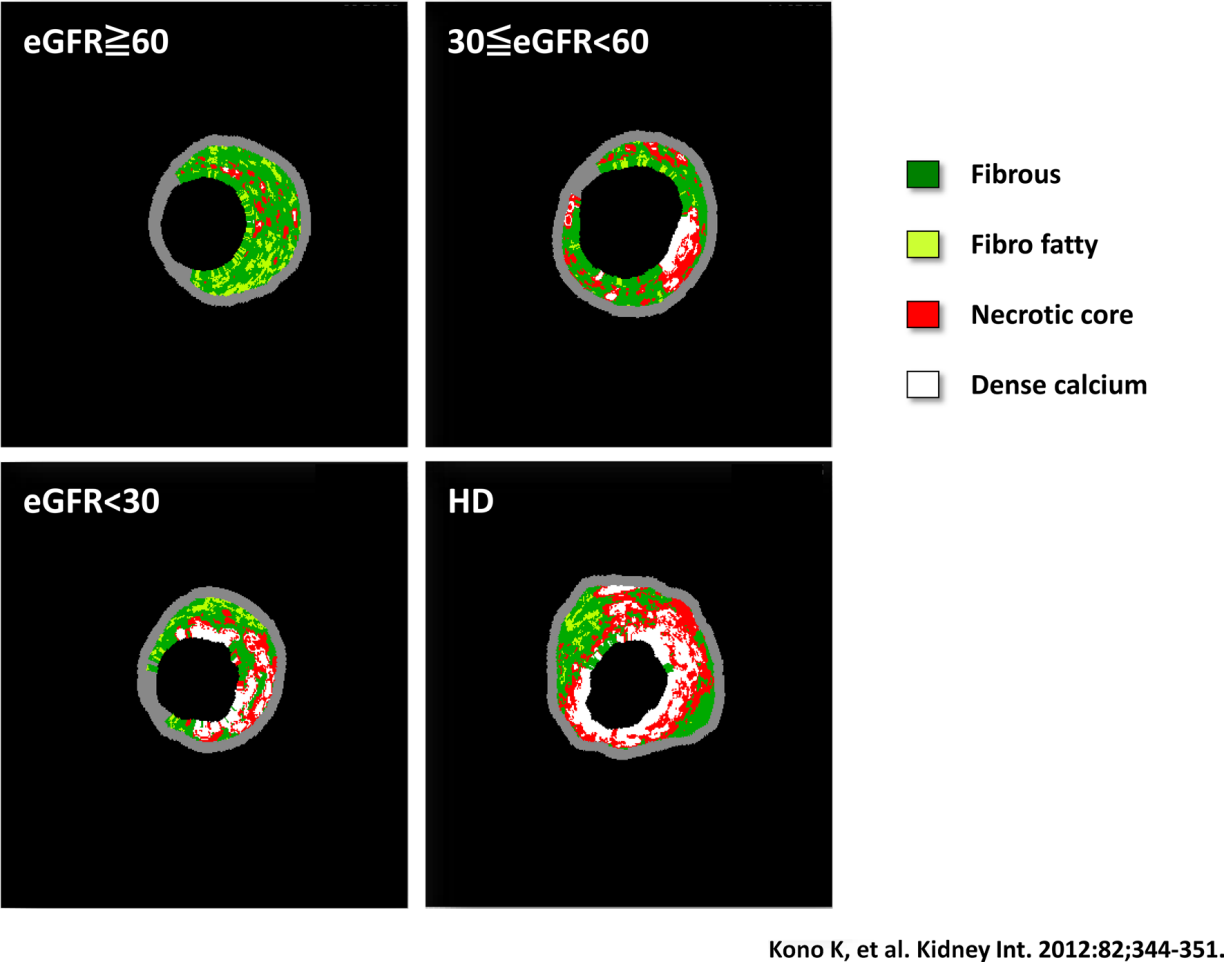
Visualized images of the coronary plaque morphology in each CKD stage. *CKD* chronic kidney disease

Acute coronary syndrome (ACS) is a syndrome that includes AMI and unstable angina pectoris that leads to myocardial damage and death due to decreased coronary blood flow. The causes of ACS involve rupture of unstable plaque and thrombus formation in the coronary artery. The stability of plaque is reportedly related to calcification (Fig. 4) [41]. Plaque with trivial or mild calcification is unstable; however, plaque with severe calcification becomes stable. In our previous study that assessed a composition of culprit coronary lesions in patients with CKD demonstrated that the percentage of necrotic core and dense calcium significantly increased in advanced CKD and a calcific lesion was particularly prominent (Fig. 5) [42]. In agreement with the previous reports [43], our study also proved that the necrotic core/dense calcium ratio was significantly higher in patients with ACS than in those with stable angina pectoris [42].

In addition to atherosclerosis, arteriolosclerosis and endothelial dysfunction are also of critical importance in CKD. Not only significant coronary artery stenosis but also arteriolosclerosis and endothelial dysfunction result in impaired coronary microcirculation and lead to cardiac hypertrophy and myocardial fibrosis. CFR reflects severity of arteriolosclerosis and endothelial dysfunction, and CAC proved to be inversely associated with CFR [44–46]. In patients with advanced CKD including those undergoing dialysis, elevations of cardiac biomarkers such as troponin T and I, patchy myocardial fibrosis, and chronic myocardial injury are often observed [47, 48]. Such myocardial ischemia in advanced CKD is diffuse. Therefore, majority do not show typical ECG changes, such as ST elevation and abnormal Q wave in myocardial infarction (MI). We speculate that these pathophysiological mechanisms may be the underlying reason for the high incidence of congestive heart failure and type 2 myocardial infarction (non-ST elevation MI without a significant coronary artery stenosis) in advanced CKD.

## Treatment of CAD in CKD

In general, aggressive treatment for CAD involves percutaneous coronary intervention (PCI) and coronary artery bypass grafting (CABG). It is very challenging to decide which treatment is better for patients with CKD, and the strategy is controversial. A randomized-controlled trial (RCT) in patients with severe CAD demonstrated that CABG resulted in lower rates of the combined end point of major adverse cardiac or cerebrovascular events at 1 year compared to PCI [49]. Another RCT conducted on subjects with multivessel CAD and diabetes mellitus also demonstrated that CABG reduced the mortality rates and MI prevalence significantly more than PCI [50].

In contrast, limited information is available regarding this issue in CKD, because, to our knowledge, no RCT has been conducted on patients with CKD until date. The results of a previous meta-analysis that included patients with CKD except for those undergoing dialysis reported no significant differences in the mortality between the CABG and PCI groups; however, CABG was superior to PCI in terms of the occurrence of MI and revascularization [51]. With respect to patients undergoing dialysis, a previous observational study using multivariable adjusted proportional hazards regression and a propensity score-matched cohort have revealed that, as compared to PCI, CABG was associated with significantly lower risks for both, death and the composite of death or MI [52]. A meta-analysis has shown that the short-term mortality is higher and the long-term cardiac event rate is lower in the CABG group as compared to that in the PCI group [53]. Although the meta-analysis also reported no significant differences in the long-term mortality, several critical issues were included in this study. First, these data are mainly from foreign countries other than Japan. Japanese patients undergoing dialysis are reported to have much better prognosis than patients undergoing dialysis in the other countries [54]. If an intervention study that targets patients with CAD undergoing dialysis is performed in Japan, favorable results can be obtained. Second, PCI is a treatment for a local vascular lesion, and CABG is a treatment for the total vessel. Patients undergoing dialysis have severe vascular calcification; therefore, cardiologists commonly encounter coronary lesions for which PCI is challenging in the clinical setting. In severe CAC, stent delivery for a target lesion is often disturbed. Furthermore, patients undergoing dialysis commonly exhibit multivessel lesions. Thus, we believe that CABG should be selected for patients undergoing dialysis if the patient condition is relatively stable.

Taking these findings into account, we propose the following indications of PCI for patients with CKD; (1) an emergency case, (2) early-to-moderate stage CKD, (3) high risk involved in surgical approach, (4) short expected life span, and (5) contraindication for CABG (single-vessel disease or two-vessel disease except for left anterior descending and/or left main trunk).

## Conclusion

The course of CAD in patients with CKD is different from that in those without CKD. Thus, occasionally, common protocol followed for patients without CKD may not be applicable to those with CKD. Furthermore, the available data regarding CKD have been derived from clinical studies that did not involve patients with CKD. Therefore, these data may not applicable to patients with CKD. Thus, the treatment decision for patients with CKD should be made after considering the patient’s clinical characteristics.
